# Self-Reported Negative Influence of Gambling Advertising in a Swedish Population-Based Sample

**DOI:** 10.1007/s10899-018-9791-x

**Published:** 2018-07-06

**Authors:** Per Binde, Ulla Romild

**Affiliations:** 1 School of Global Studies, University of Gothenburg, PO Box 700, 40530 Gothenburg, Sweden; 2 Public Health Agency of Sweden, PO Box 505, 83126 Östersund, Sweden

**Keywords:** Gambling, Problem gambling, Marketing, Promotion, Advertising

## Abstract

This study investigated the negative influence of gambling advertising, that is, gambling more often or for more money than intended. We analyzed data from wave four of the Swedish Longitudinal Gambling Study (Swelogs), in which the self-perceived negative influence of gambling advertising was measured by responses to three survey questions. Few gamblers reported having been negatively influenced by gambling advertising. Among those who reported such influence, problem gamblers were overrepresented. Those who had set limits for their gambling reported a negative influence from advertising more often than others, which likely was caused by a perception that advertising is detrimental to efforts to cut down on excessive gambling. A multivariate regression analysis showed that negative influence from gambling advertising was positively associated with problem gambling, gambling at least monthly, participation in online gambling, and being in the age group 30–49 years. We conclude that although few gamblers are negatively influenced by gambling advertising, the adverse effects on those that are should not be neglected. For a considerable number of people, gambling advertising substantially contributes to problem gambling.

## Introduction

Gambling advertising is intended to stimulate people to gamble. It typically portrays gambling in a positive light, associating it with having fun, getting a thrill, winning money, being knowledgeable about sports and horse racing, and having the chance to become rich. In many countries, including Sweden, marketing, sponsoring, and advertising of gambling is ubiquitous, appearing in television, on the Internet, in social media, and in virtually all other media channels.

Marketing for potentially addictive consumer goods, such as alcoholic beverages and tobacco, are in many countries subject to restrictions because it is considered detrimental to public health to stimulate demand for such goods. Gambling disorder is classified in the fifth edition of the Diagnostic and Statistical Manual of Mental Disorders (DSM-5) as an addiction disorder (American Psychiatric Association 2013), and the wider concept of problem gambling has aptly been defined as ‘difficulties in limiting money and/or time spent on gambling which leads to adverse consequences for the gambler, others, or for the community’ (Gambling Research Australia 2005). There are therefore good reasons to ask if gambling advertising, which seldom is as strictly restricted as alcohol and tobacco advertising is, might have the effect of stimulating the demand for gambling to excessive levels. It is also relevant to ask if particular kinds of advertising are more likely than others to lead to excessive gambling, what types of gamblers are most vulnerable to its negative impact, and what kinds of messages are most risky in these respects.

There is limited empirical evidence available for answering these questions. It is methodologically challenging to assess the impact of advertising on consumer behavior, and this might dissuade research on gambling advertising. It is difficult to produce hard evidence, and results might be indicative only. Nevertheless, the number of studies is steadily growing. There were at least 33 empirical studies focusing on gambling advertising as of April 2014 (Binde 2014a), and since then (up to October 2017) the results of about 40 additional studies have been presented (Binde 2017).

In this article, we contribute to the growing knowledge about the impacts of gambling advertising by presenting results and analyses from a study on self-perceived negative influence of gambling advertising in a Swedish population-based sample. Our research questions were the following.

1. How common is it that gamblers report a negative influence of gambling advertising?
2. What characterizes those who report such influence?
3. Is there any specific kind of advertising that more than others leads gamblers to report negative influence of gambling advertising?

A sketch of the Swedish gambling scene will help to situate this study in its national context (for a more detailed picture, see Binde 2014b; Jonsson and Rönnberg 2009). In 2014, the year in which the data used in this study were collected, land-based gambling was offered either by state owned or state-controlled companies or by lotteries run by non-profit organizations serving the public good. About half of the online gambling market belonged to foreign-based companies without a license in Sweden. These companies were the only ones who offered online casino games and slots, forms of gambling that no licensed companies were allowed to offer (except online poker). In 2014, among the gamblers who called the national problem gambling help-line (and for whom information on the main form of gambling causing problems was recorded), 43% had problems specifically with online casino games; a further 10% had problems with online poker and 13% with online sports betting (Folkhälsomyndigheten 2015).

Swedish law forbids anyone to promote for commercial purposes gambling offered by companies without a license in Sweden. However, this law has been largely ineffective over the past decade because of legal technicalities and because Swedish authorities have no influence on the content of commercial television broadcasts from abroad. In 2014, about three quarters of all gambling advertising in Sweden was made by unlicensed companies (Lotteriinspektionen 2015). The total volume of gambling advertising increased steadily from 2000 to 2014, with an almost exponential growth from 2011. The gross advertising expenses in 2014 (before rebates) were nearly three times as high compared to 2010 (SEK 3.7 billion and SEK 1.3 billion, respectively). In the public debate, the amount of gambling advertising is often described as excessive, and it is feared that the intense marketing activity is contributing to problem gambling.

## Literature Review

A number of previous articles and reports have discussed the effects of gambling advertising and have reviewed the relevant literature (Binde 2007a; Clotfelter and Cook 1989; Clotfelter et al. 1999; Griffiths 2005; Parke et al. 2015; Planzer and Wardle 2011; Schottler Consulting 2012; Zangeneh et al. 2008). The most comprehensive of such publications is a research review from 2014, written by one of the current authors, that examined most of the empirical studies of gambling advertising and summed up the state of knowledge (Binde 2014a). The overview of previous research offered here is based on the findings of that review.

As mentioned, measuring the effect of advertising on consumers is a methodologically challenging endeavor. Some advertising content is deliberately created so as to bypass critical and rational evaluation. In suggestive and implicit ways, advertising might shift consumers’ perceptions of a particular product or offer in a more favorable direction. Advertising’s impact on the individual level therefore needs not be great for advertising to be profitable for companies because it suffices, on the population level, that only a small fraction of consumers are made more willing to buy the products offered or have their preferences slightly shifted so that they choose, the next time they make a purchase, the advertised product rather than a similar product from another company. On the population level, advertising efficiency can be measured by comparing the volume of advertising to the volume of sales over time.

This said, there are certainly circumstances under which consumers can recall perfectly well how specific advertisements have influenced their purchases. For example, an individual might view an advertisement for a trotting pool jackpot in an online edition of a newspaper and decide to take advantage of the extra money offered to winners by making a bet. The person will be perfectly aware of the connection between the advertisement and his or her gambling behavior and likely to remember it for some time afterwards. If such events are repeated, the individual is likely to be aware of advertising having a substantial impact on participation in gambling. More generally, awareness of advertising’s impact is greater when the message is a particular offer compared to when advertising suggests the excellence of a brand or the positive image of a company. Advertising research has shown that brand advertising is more effective when it downplays factual content, appeals to emotions, and is processed by consumers at a low level of attention (Heath et al. 2006).

Consumers can also tell how they perceive themselves to be influenced by advertising messages, although this is subject to well-known biases included in the concept of the ‘third person effect’, that is, people tend to underestimate the influence of advertising on themselves and to overestimate its effect on others (Davison 1983; Jensen and Collins 2008; Johnston and Bourgeois 2015). Furthermore, consumers can, when prompted, estimate their exposure to advertising of various kinds, although such estimates are likely to be biased by recall errors, varying interest in the products marketed, and other personal and psychological factors. Thus, the ability of consumers to report advertising’s influence on their perceptions and behaviors varies between poor and good, and the methodological challenges in measuring the influence might be significant.

An approach to studying the effects of gambling advertising on gambling participation and problem gambling often used in previous research is to ask a sample of gamblers, using questionnaires or qualitative interviews, about their participation in gambling, their problems with gambling, their exposure to gambling advertising, the self-perceived influence of such exposure, and other factors assumed to be relevant.

In almost all such studies, the majority of participants have claimed that gambling advertising has no effect on their participation in gambling. For example, a Danish study found that 78% of the respondents reported that advertising had no importance for their consumption of gambling, 19% reported small or minor importance, and only 2% reported that advertising had a big or very big importance (Bjerg Kommunikation 2014). Two other Scandinavian studies have arrived at similar conclusions (Fridberg and Fels Birkelund 2016; Pallesen et al. 2016).

These two Scandinavian studies also showed that many or the majority of respondents reported that gambling advertising increased their interest in gambling as well as their knowledge about gambling products and companies (Fridberg and Fels Birkelund 2016; Pallesen et al. 2016). However, this did not necessarily lead to favorable attitudes towards gambling in general, specific games, or particular companies. The two studies showed that only 8–12% of respondents agreed strongly or somewhat to the statement ‘I think more positively about gambling because of gambling advertisements’. The majority of respondents (54–69%) disagreed strongly with this statement.

Numerous cross-sectional studies have shown that problem gamblers recall having seen more gambling advertising than non-problem gamblers (e.g., Clemens et al. 2017; Fried et al. 2010). The interpretation of this result might be that high exposure to gambling advertising has contributed to their problems, that problem gamblers are more attentive to gambling advertising because they are already highly involved in gambling, that a third confounding factor explains the relationship, or that the relationship is created by a combination of the aforementioned circumstances.

Another recurrent result in previous studies is that problem gamblers report greater influence of advertising on their gambling involvement than non-problem gamblers (e.g., Gainsbury et al. 2016; Hanss et al. 2015). Qualitative studies have shed light on this association by showing that some problem gamblers perceive that advertising gives them hard to resist impulses to gamble (Binde 2009; Hing et al. 2014). They realize that their gambling is excessive, but advertising contributes to impaired control over their gambling. Advertising worsens their problems by making them gamble more than intended and might provoke relapse among those who wish to abstain from gambling, as has been suggested also by quantitative studies (Grant and Kim 2001; Hing et al. 2015).

In three previous studies, respondents were asked specifically if gambling advertising had made them gamble more than intended or really wanted to, and these studies show that problem gamblers answer in the affirmative to such questions more often than non-problem gamblers (Hing et al. 2015, 2017a; Schottler Consulting 2012).

With regard to other sub-groups of gamblers and types of games, a study from New Zealand found that self-reported influence of gambling advertising varied across ethnic groups (Clarke et al. 2006), while another study from the same country found that the self-rated impact of advertising differed between various forms of gambling (Schottler Consulting 2012). The latter study also found that problem gamblers reported a stronger impact from advertising slogans about big jackpots than non-problem gamblers.

It is likely that advertising messages that connect with risk factors for problem gambling—such as cognitive errors regarding the chances of winning, ideas that gambling is an easy way to make money, or using gambling as an escape from personal troubles—are more harmful than other messages (Binde 2007a; McMullan and Miller 2010; Schottler Consulting 2012). However, this has not yet been demonstrated in empirical studies. Nevertheless, ethical codes and regulations of gambling advertising typically prohibit such advertising messages (e.g., CAP and BCAP 2014; IGRG 2015).

In summary, self-report methods have been used in numerous studies of the impact of gambling advertising, but there are questions regarding the ability of respondents to accurately recall to what extent they have been exposed to advertising and how they are influenced by it. It has been concluded, however, that data from self-reporting become more valuable when answers from various types of gamblers and regarding various forms of advertising are compared, i.e., when the analysis focuses on the relative rather than the absolute impact (Binde 2014a). The study reported here is of such a comparative kind.

## Methods

### Data

The dataset analyzed was wave four in the epidemiological track (EP4) of the Swedish longitudinal gambling study (Swelogs). The main objective of Swelogs was to longitudinally and prospectively analyze factors relating to the prevalence and incidence of problem gambling. The first wave of Swelogs (EP1) was conducted in 2008–2009. A random but stratified selection from the Swedish National Register of the total population, consisting of 15,000 individuals between the ages of 16 and 84, were contacted. Slightly more than 8000 individuals participated in the first wave. These individuals, except for those who had declined further participation, were re-contacted over the following 5 years for three new interviews. Register data on demographic and socioeconomic variables pertaining to the respondents were obtained from Statistics Sweden. Details on the Swelogs study design and methodology have been published elsewhere (Romild et al. 2014).

EP4 data were collected in 2014. About 7000 individuals aged 22–90 years were contacted, out of which 3559 responded. This study concerns the 2163 respondents who had gambled at least once in the past 12 months and were asked the questions on self-perceived negative influence of gambling advertising.

The longitudinal design of Swelogs means that the participants in EP4 came from a randomly selected sample of the adult Swedish population and that the individuals had all chosen to repeatedly participate in the study. The original sampling strategy included oversampling of young people and groups with estimated elevated risk of gambling problems, but these are also the groups with higher attrition, and by the time of EP4 all participants were aged 22 or older and the proportions of socio-demographic variables, used for stratification, among the remaining respondents had shrunk towards the proportions in the population. Men were more likely to participate in Swelogs initially, but attrition was slightly higher among men than among women in waves 3 and 4.

### Measurements

Three questions in the EP4 interview concerned self-perceived negative influence of gambling advertising. In this article, these will be called the Negative Influence of Gambling Advertising (NIGA) questions. The questions were formulated ad hoc to elicit information about aspects of gambling advertising of interest in a public health perspective. As far as we know, there is currently no evaluated or widely used instrument or questionnaire for measuring perceptions of and attitudes to gambling advertising, although the Effects of Gambling Advertising Questionnaire (EGAQ) has been used, in full or part, in at least three studies (Derevensky et al. 2010; Fridberg and Fels Birkelund 2016; Hanss et al. 2015). Questions similar to the NIGA questions—i.e. asking about negative influence on gambling behavior—have been used in three previous studies of gambling marketing (Hing et al. 2015, 2017a; Schottler Consulting 2012).

The first of the NIGA questions was ‘Have you been influenced by advertising for big jackpots to gamble more often or for more money than you intended?’ The other two questions were similar, but instead of asking about ‘advertising for big jackpots’, they were about ‘advertising for bonuses for online gambling’ and ‘gambling advertising in general’. The respondent could choose to answer ‘No, never’, ‘Yes, sometimes’, ‘Yes, several times’ and ‘Don’t know’. The timeframe of the query was the past year.

The particular phrasing of the questions—‘gamble more often or for more money than intended’—was chosen to elicit responses regarding self-perceived negative influence of gambling advertising. Gambling advertising might stimulate people to gamble moderately within reasonable limits or choose one gambling service or offer rather than another. Such harmless—at least in the short-term perspective—influence of gambling advertising was not of interest in the public health perspective of the EP4 study.

Because of the ad-hoc nature of the NIGA questions, they should not be considered to be a psychometric instrument, which would have required the operationalization of concepts and thereafter verification of validity and reliability. However, we performed an exploratory factor analysis, which showed that the NIGA questions are related to one single factor. Factor loadings (principal components) ranged between 0.66 and 0.82. The parallel model assumption, suggesting equal variances for all items, was rejected. Cronbach’s alpha equaled 0.57. The strongest item is the third question regarding influenced in general by gambling advertisements, without which the alpha estimate is reduced to 0.29. Inter-item correlations range between 0.19 and 0.41, where the weakest is between the two first questions concerning bonuses and jackpots.

Problem gambling was measured in the Swelogs epidemiological studies by the Problem Gambling Severity Index (PGSI, Ferris and Wynne 2001).

A number of demographic measures (register data supplied by Statistics Sweden) were used in a regression analysis (see Table 3), and we also analyzed the responses to a survey question about respondents’ self-imposed limits on gambling (see Fig. 3).

### Analyses

The EP4 data can be analyzed either weighted—that is, statistically adjusted to resemble the population in general—or unweighted. In this article, unweighted data were used, but all significance tests were confirmed with weighted data.

The NIGA questions were not included in EP1, EP2, or EP3 of Swelogs. Therefore, we could only perform a cross-sectional analysis and not a prospective one.

## Results

### The Extent of Self-Perceived Negative Advertising Influence

**Fig. 1:**
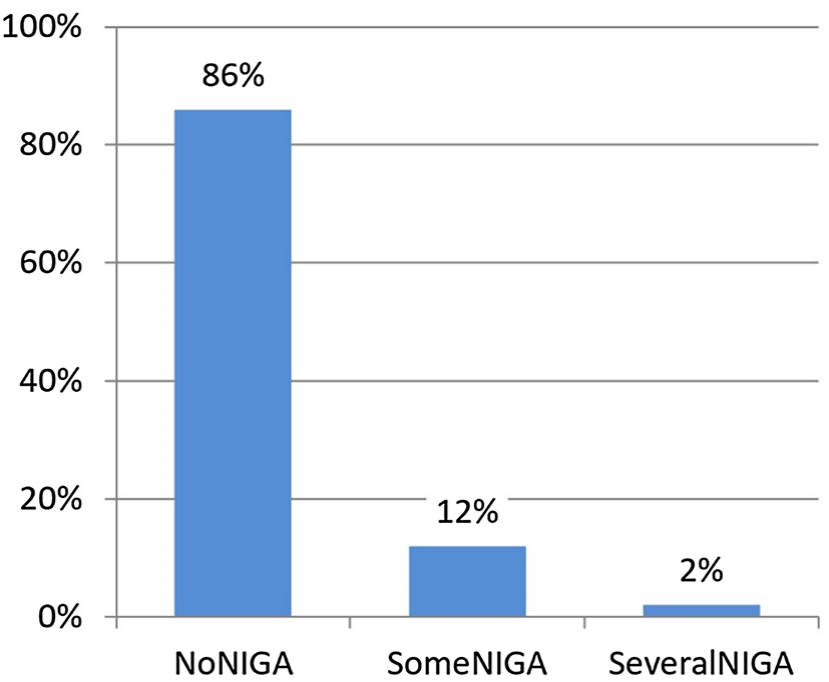
Merged responses to the NIGA questions

The responses to the NIGA questions are presented in Table 1. In order to facilitate the analysis, the responses were merged (Fig. 1). Those who answered in the negative to all three questions were termed ‘No Negative Influence from Gambling Advertising’ (NoNIGA), those who answered ‘Yes, sometimes’, but not ‘Yes, several times’, to one or more of the three questions were termed ‘Some Negative Influence from Gambling Advertising’ (SomeNIGA) and those who answered ‘Yes, several times’ to one or more of the three questions were termed ‘Several Times Negatively Influenced by Gambling Advertising’ (SeveralNIGA).

**Table 1:** Response distributions for the NIGA questions

Have you been influenced by … to gamble more often or for more money than you intended?	No, never	Yes, sometimes	Yes, several times	Don’t know	No answer
… advertising for big jackpots …	1904 (88.0%)	159 (7.4%)	26 (1.2%)	2 (0.1%)	72 (3.3%)
… advertising for bonuses for online gambling …	2021 (93.4%)	52 (2.4%)	11 (0.5%)	4 (0.2)	75 (3.5%)
… gambling advertising in general …	1962 (90.7%)	101 (4.7%)	16 (0.7%)	9 (0.4%)	75 (3.5%)

The results presented in Fig. 1 show that the overwhelming majority of respondents (86%) perceived no negative impact of advertising on their gambling. Occasional negative influence was reported by 12% of the sample, and negative influence on several occasions was reported by around 2%. It should be noted (in Table 1) that fewer than 1% of the respondents answered ‘Don’t know’ to the NIGA questions, and only around 3% did not answer them, which indicates that a very large majority found it unproblematic to judge the extent of advertising’s impact when asked about it.

### Type of Advertising

Table 1 shows that, among the three response alternatives offered, advertising for jackpots was reported to have the greatest negative influence, followed by advertising in general and then by advertising for bonuses in online gambling.

Advertising for bonuses is only made by gambling companies without a license in Sweden because no licensed company is allowed to offer bonuses. We investigated this kind of advertising more in detail. In the sample, only 228 individuals (6%) had gambled with companies without a license in Sweden. Among these, 38 individuals reported a negative influence of advertising for bonuses, that is, 17%. Thus, bonus advertising made by online companies without a license in Sweden seems to have been perceived by a relatively large number of those who were customers of such companies to have a negative influence.

### Problem Gambling

**Fig. 2:**
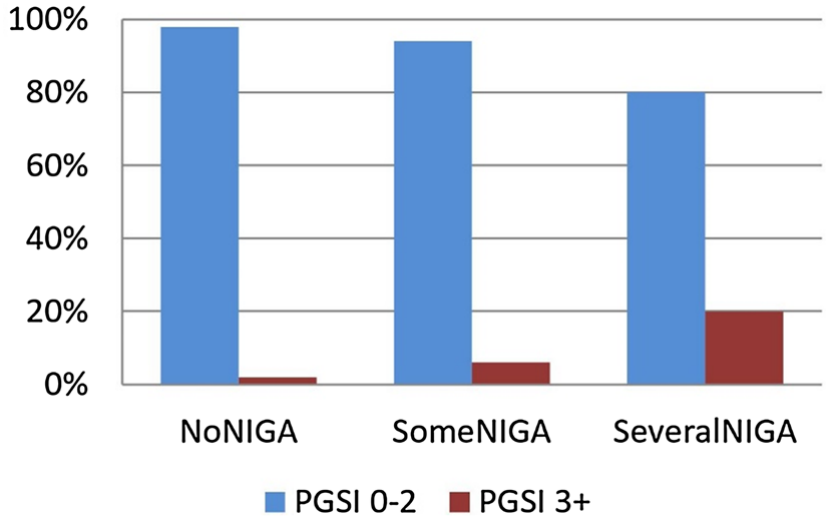
Responses to NIGA question in relation to PGSI scores

The distribution of perceived negative influence of gambling advertising in relation to PGSI score is shown in Fig. 2 and Table 2. Of the past-year gamblers studied here, 2.6% (56 individuals) were classified as being moderate risk or problem gamblers (PGSI 3+). Figure 2 shows the relationship between being a PGSI 3+ gambler or not and self-perceived NIGA. One-fifth (19.5%) of those who perceived that they several times had been negatively influenced by gambling advertising (SeveralNIGA) scored three or more points on the PGSI, compared to only 1% among those who perceived no negative influence. The relation between the total PGSI score and the self-perceived NIGA was significant, with Kendall’s tau-b equal to .25 (*p* < .01).

**Table 2:** Cross-tabulation of responses to the NIGA questions in relation to PGSI scores

PGSI	NoNIGA	SomeNIGA	SeveralNIGA
0	1682 (89%)	189 (10%)	25 (1%)
1–2	82 (59%)	49 (36%)	8 (6%)
3–4	22 (61%)	11 (31%)	3 (8%)
5–7	2 (25%)	2 (25%)	4 (50%)
8+	8 (73%)	2 (18%)	1 (9%)

Table 2 shows the distribution of PGSI scores in relation to responses to the NIGA questions. It should be noted that the *n*-values are very small in some of the cells, and thus the exact proportions within the cells might be affected by random errors. Differences in the distribution of NIGA between the five different PGSI categories was significant according to the Kruskal–Wallis test (χ^2^ = 145.4; df = 4; *p* < .001). Pairwise comparisons using the Mann–Whitney test between the different categories showed that the group with no elevated risk of gambling problems (PGSI = 0) was significantly different (*p* < .001) from all other groups except the PGSI 8+ group (Asymp z = − 1.73; *p* = .083). Similarly, the PGSI 5–7 group was different from all other groups, including the PGSI 8+ group (Asymp z = − 2.18; *p* = .030). The results indicate a trend that the self-perceived negative impact of gambling advertising becomes greater with increasing problem gambling severity, except for the very severe cases, that is, PGSI 8+. In this group, only 1 of the 11 respondents reported SeveralNIGA.

### Socio-demographic and Gambling Behavior Variables

We performed a multivariate logistic regression analysis in order to explore the relationships between NIGA (NoNIGA compared to SomeNIGA/SeveralNIGA combined) and a number of socio-demographic and gambling behavior variables (Table 3).

**Table 3:** Multivariate regression analysis of the association between the dichotomous NIGA variable and selected demographic and gambling behavior variables

	Crude OR	95% CI	Adj. OR	95% CI
Gender				
Female (ref)	1.00		1.00	
Male	*1.67*	*1.28*–*2.19*	1.35	1.00–1.82
Age				
≤ 29	*2.44*	*1.51*–*3.96*	1.63	.96–2.78
30–39	*3.16*	*1.94*–*5.14*	*2.43*	*1.43*–*4.13*
40–49	*4.84*	*2.84*–*8.26*	*3.66*	*2.05*–*6.52*
50–59	*2.19*	*1.17*–*4.10*	1.67	.87–3.23
60–69	1.56	.80–3.01	1.36	.69–2.68
70+ (ref)	1.00		1.00	
Country of birth				
Sweden (ref)	1.00		1.00	
Europe, outside Sweden	1.39	.92–2.11	1.18	.75–1.87
Outside Europe	*1.90*	*1.27*–*2.86*	1.38	.88–2.19
Education				
Elementary school (ref)	1.00		1.00	
High school	1.40	.91–2.16	1.08	.66–1.74
Higher education/university	*1.63*	*1.04*–*2.53*	1.44	.87–2.36
PGSI				
0 (ref)	1.00		1.00	
1–2	*5.46*	*3.79*–*7.88*	*4.00*	*2.65*–*6.03*
3+	*5.65*	*3.24*–*9.83*	*3.50*	*1.91*–*6.42*
Gambling				
Gambled a few times (ref)	1.00		1.00	
Gambled at least monthly	*1.97*	*1.54*–*2.53*	*1.61*	*1.162*–*2.23*
Gambling online				
Not at all in the past year (ref)	1.00		1.00	
Gambled a few times	*3.24*	*2.36*–*4.45*	*2.56*	*1.81*–*3.63*
Gambled at least monthly	*4.51*	*3.32*–*6.14*	*2.30*	*1.57*–*3.38*
Nagelkerke R^2^			.178	
Hosmer–Lemeshow test			.052	

Crude and adjusted odds ratios (ORs). Italics indicate statistically significant relationships (*p* < .05)

The adjusted odds ratios of the regression analysis showed that PGSI score had the strongest association with the NIGA variable. Monthly or more frequent gambling, having gambled online, and being in the age group 30–49 years were also associated with NIGA. The model explained 18% of the variation according to the Nagelkerke R^2^, and the Hosmer–Lemeshow test showed that the model is a possible representation of the data (*p* = .052). Gender, country of birth, and education were not significantly associated with NIGA in this regression model. However, the crude and unadjusted odds ratios showed that being male, being born outside of Europe, and having higher education were significantly associated with NIGA. These relationships disappeared in the regression analysis because they were secondary to the significant factors in the model.

### Self-Imposed Limits on Gambling

We compared the results from the NIGA questions with the respondents’ answers to the following question in the EP4 questionnaire: ‘Do you do anything in particular to limit the amount of money or time that you spend on gambling?’.

**Fig. 3:**
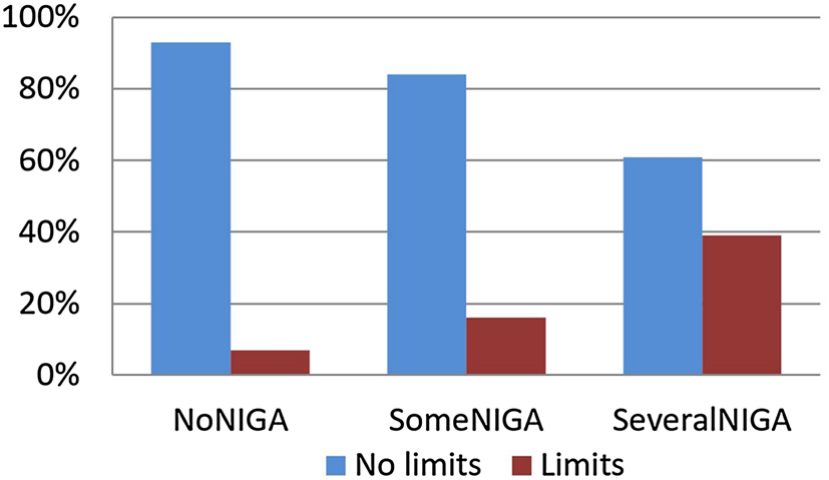
Self-imposed limits on gambling in relation to perceived negative impact of gambling advertising

We found a significant association (*p* < .001) between self-imposed limits on the amount of money and time spent on gambling and self-perceived negative impact of gambling advertising (Fig. 3) using Pearson’s chi-squared test (χ^2^ = 71.1; df = 2). About one-third (39%) of the SeveralNIGA respondents and about one-sixth (16%) of the SomeNIGA respondents had set self-imposed limits on their gambling compared to only 7% of NoNIGA respondents.

## Concluding Discussion

We analyzed a large dataset with 2163 participants from the general population who had gambled at least once in the past 12 months. Our first research question was: how common is it that gamblers report negative influence of gambling advertising? We found that few gamblers—only about 2%—reported repeated negative influence of gambling advertising (SeveralNIGA).

Our second research question was: what characterizes those who report negative influence from gambling advertising? We found that among those who report NIGA, moderate risk and problem gamblers (PGSI 3+) were overrepresented; about 20% of those who reported repeated NIGA. This finding is in line with results from previous studies. Gambling advertising may contribute to problem gambling, and problem gamblers are more sensitive to advertising impact than non-problem gamblers.

The distribution of NIGA responses across PGSI levels suggests that the more severe the gambling problems are, the greater the NIGA, except for the most severe cases. This result is consistent with observations from a previous study (Binde 2009). That study reviewed research on the perceived impact of gambling advertising and found that the higher the average problem gambling score in the study populations, the greater the impact. However, that study’s qualitative interviews showed that people with severe gambling problems were already so excessively involved in gambling that advertising typically meant little to them.

We found a strong association between NIGA and self-imposed limits on the amount of time and money spent on gambling. About 1 in 3 of those who reported repeated NIGA had set such limits. The most likely reason for this association is that exposure to gambling advertising gave these individuals hard to resist impulses to gamble and therefore was detrimental to their efforts to keep gambling within what they considered to be reasonable limits. As far as we know, this is the first quantitative investigation that shows that gambling advertising in this way maintains or risks maintaining already existing gambling problems.

The multivariate regression analysis showed that NIGA was positively associated with PGSI score, gambling monthly or more often, participating in online gambling, and being in the age group 30–49 years. This result can be interpreted as indicating that being middle aged, gambling problematically, gambling often, and gambling on heavily advertised and risky forms of gambling is the characteristic profile of the individual reporting NIGA. In Sweden, at the time of the study, the most advertised forms of gambling was online casino (including online slots) and online sports betting, which also were the types of gambling that the majority of callers to the national help-line reported as causing them problems (Folkhälsomyndigheten 2015). Although in raw numbers males, those born outside of Europe, and people with higher education are overrepresented among those who report NIGA, the regression analysis showed that these variables were secondary to the gambling behavior variables. The only enigmatic result in this study is the overrepresentation of people with a higher education among those who report NIGA. We speculate that this might be caused by such people being more active and informed gambling consumers than others and being more interested in taking advantage of offers and acquiring various benefits. This might make them more attentive to advertising offers, and also more disappointed, and likely to report their disappointment in a survey like the Swelogs EP4, if the offers have made them gamble more than they had intended, presumably without having won or otherwise benefited satisfactorily from the offers.

With regard to our third research question—the impact of different kinds of advertising—we found that advertising for big jackpots was more often reported to have a negative influence than the other two kinds of advertising mentioned in the questionnaire. While many types of gamblers are likely to be attracted by having the chance to hit a big jackpot, problem gamblers might be especially vulnerable to this advertising message. They might have a desperate hope that winning big would allow them to solve the economic problems caused by heavy gambling and to recoup their losses. Furthermore, as one interviewee in a qualitative study on gambling advertising impact said, jackpot advertising might more generally stimulate one to excessive gambling:

‘… you see the advertising, and then you gamble even more. [You say to yourself:] – Heck, there’s a jackpot, now I must really push it to the limit!’ (Binde 2007b, p. 63, translated)

Because relatively few Swedes were, at the time of the study, customers of foreign-based gambling companies without licenses in Sweden, advertising for bonuses in online gambling—which is made only by these companies—was not mentioned as often as jackpots. However, of those few in the sample who were such customers, 17% reported negative influences from advertising for bonuses. This relatively high figure indicates that such advertising is quite problematic for a specific segment of online gamblers who are attracted by sports betting offers, as well as online casino and slots, which in Sweden are not offered by licensed operators.

How concerned should we be by the findings of this study? On the one hand, very few gamblers perceived themselves to be repeatedly negatively influenced by gambling advertising. Among the few who did, this study cannot tell how harmful that influence was. Because most of the PGSI 3+ SeveralNIGA respondents were ‘moderate risk gamblers’, it is likely that in most cases the influence was only marginally harmful. There is no evidence in this study that gambling advertising in mass media substantially contributes to gambling problems.

On the other hand, a substantial portion of the PGSI 3+ respondents reported SeveralNIGA (see Table 2). In Sweden in 2015 (with a population close to 10 million), there were about 134,000 moderate risk and problem gamblers. This means that tens of thousands of these were repeatedly influenced by gambling advertising to gamble more often and for more money than they intended. This adverse consequence should not be neglected. Among these people, gambling advertising worsens problems and adds to the harm caused by excessive gambling.

The strength of this study is the relatively large population-based sample with more than 2000 respondents, a reasonably high response rate, and reliable socio-demographic registry variables from Statistics Sweden. The questions in the survey were carefully chosen to capture as accurately as possible the self-perceived negative influence of gambling advertising.

A limitation of the study is that NIGA is a self-report variable. Individuals might not accurately recall advertising’s influence or correctly judge its impact on their gambling behavior. It should be noted, however, that remarkably few respondents—fewer than 1%—reported that they were unsure of the extent, if any, to which they had been directly and negatively influenced by gambling advertising, in this case ‘gambling more often or for more money than intended’, and a further 3% did not answer the question. This suggests that most respondents had an idea of how much time and money they are willing to spend on gambling and that almost all believed that they could judge if advertising had made them spend more than that. Nevertheless, some respondents might have been unaware of gambling advertising having a negative influence on the extent of their gambling because the advertising only gave them subtle cues and suggestions. Furthermore, the NIGA questions are not a psychometric instrument. Little is known about their validity and reliability, except for the results of our exploratory factor analysis, which showed that the NIGA questions are related to one single factor. However, should such a psychometric instrument be developed, the results of this study might be of value.

Another limitation of the study is that because Swelogs EP4 was the fourth wave in a longitudinal study, the sample was not fully representative of the Swedish population. The youngest in the sample had become at least 22 years of age. In addition, respondents were only asked about two specific types of advertising in addition to gambling advertising in general. Preferably, the questions should have included many types of gambling advertising, messages, and inducements (Hing et al. 2017b). The NIGA questions concerned only ‘gambling advertising’, which was likely to have been understood by most respondents as mass media advertising, and thus promotional messages sent by gambling companies to their customers were not covered by the study. The number of PGSI 3+ and SeveralNIGA respondents were relatively few, which decreased the precision in the statistical analyses. It is possible that some people with gambling problems exaggerate the influence of gambling advertising on their behavior because they feel anger over the way gambling companies promote their products. However, a qualitative study of 25 Swedish problem gamblers found no relationship between attitudes towards gambling advertising and self-perceived negative advertising influence (Binde 2009).

Future research should study the self-perceived negative influence of gambling advertising more in depth by exploring its associations with variables such as the media channels through which promotional messages are sent and attitudes towards gambling and gambling advertising. Of specific interest would be marketing in social media, which is used by gambling companies in order to reach potential and existing customers in a more direct and personal way (e.g., Abarbanel et al. 2017; Gainsbury et al. 2016).

This study has several policy implications. Gambling companies—in particular online operators who have no physical venues that provide a point of contact with existing or potential customers—rely heavily on advertising and other forms of promotion to recruit customers and stimulate sales. The stiff competition in the online market means that investing heavily in advertising is a must for most companies. Gambling companies and regulatory authorities should take measures to prevent advertising and promotions targeting or reaching people who have a problem with gambling. For example, online gambling companies that use behavioral tracking tools should stop sending commercial messages to customers who are indicated as having risky or problematic gambling behavior. As to the content of advertising, special restraint and moderation should be shown when formulating promotional messages about big jackpots and bonuses. Although the effectiveness of social marketing and public education seems to be low to moderate in preventing harmful gambling (Williams et al. 2012), such measures could be considered as a response to commercial marketing (Hing et al. 2017a; Lemarié and Chebat 2013).

## References

[CR1] Abarbanel B, Gainsbury SM, King D, Hing N, Delfabbro PH (2017). Gambling games on social platforms: How do advertisements for social casino games target young adults?. Policy & Internet.

[CR2] American Psychiatric Association (2013). Diagnostic and Statistical Manual of Mental Disorders.

[CR3] Binde P (2007). Selling dreams—Causing nightmares? On gambling advertising and problem gambling. Journal of Gambling Issues.

[CR4] Binde, P. (2007b). *Spelreklam och spelberoende: En intervjustudie* [Gambling advertising and problem gambling: An interview study]. Östersund: Statens folkhälsoinstitut.

[CR5] Binde P (2009). Exploring the impact of gambling advertising: An interview study of problem gamblers. International Journal of Mental Health and Addiction.

[CR6] Binde P (2014). Gambling advertising: A critical research review.

[CR7] Binde P (2014). Gambling in Sweden: The cultural and socio-political context. Addiction.

[CR8] Binde, P. (2017). *A bibliography of empirical studies on gambling advertising* (1st ed.). Gothenburg, Sweden: OnGambling.org. https://ongambling.org/bibliography-gambling-advertising.pdf

[CR9] Bjerg Kommunikation. (2014). *Befolkningsundersøgelse: Danskernes spil om penge 2014* [Population study: Gambling among the Danes in 2014]. København: Author.

[CR10] CAP & BCAP (2014). Help note: Guidance on the rules for gambling advertisements.

[CR11] Clarke D, Tse S, Abbott M, Townsend S, Kingi P, Manaia W (2006). Key indicators of the transition from social to problem gambling. International Journal of Mental Health and Addiction.

[CR12] Clemens F, Hanewinkel R, Morgenstern M (2017). Exposure to gambling advertisements and gambling behavior in young people. Journal of Gambling Studies.

[CR13] Clotfelter CT, Cook PJ (1989). Selling hope: State lotteries in America.

[CR14] Clotfelter CT, Cook PJ, Edell JA, Moore M (1999). State lotteries at the turn of the century. Report to the National Gambling Impact Study Commission.

[CR15] Davison WP (1983). The third-person effect in communication. Public Opinion Quarterly.

[CR16] Derevensky J, Sklar A, Gupta R, Messerlian C (2010). An empirical study examining the impact of gambling advertisements on adolescent gambling attitudes and behaviors. International Journal of Mental Health and Addiction.

[CR17] Ferris J, Wynne H (2001). The Canadian Problem Gambling Index: Final report.

[CR18] Folkhälsomyndigheten. (2015). *Stödlinjen årsrapport 2014* [Problem gambling helpline, yearly report 2014]. Östersund: Author. Retreived from: www.folkhalsomyndigheten.se/documents/projektwebbar/spelprevention/publikationer/Stodlinjen-arsrapport-2014.pdf

[CR19] Fridberg, T., & Fels Birkelund, J. (2016). *Pengespil og spilleproblemer i Danmark 2005*–*2016* [Gambling and gambling problems in Denmark 2005–2016]. København: SFI.

[CR20] Fried BG, Teichman M, Rahav G (2010). Adolescent gambling: Temperament, sense of coherence and exposure to advertising. Addiction Research & Theory.

[CR21] Gainsbury SM, King DL, Russell AMT, Delfabbro P, Derevensky J, Hing N (2016). Exposure to and engagement with gambling marketing in social media: Reported impacts on moderate-risk and problem gamblers. Psychology of Addictive Behaviors.

[CR22] Gambling Research Australia (2005). Problem gambling and harm: Towards a national definition.

[CR23] Grant JE, Kim SW (2001). Demographic and clinical features of 131 adult pathological gamblers. Journal of Clinical Psychiatry.

[CR24] Griffiths MD (2005). Does gambling advertising contribute to problem gambling?. International Journal of Mental Health and Addiction.

[CR25] Hanss D, Mentzoni RA, Griffiths MD, Pallesen S (2015). The impact of gambling advertising: Problem gamblers report stronger impacts on involvement, knowledge, and awareness than recreational gamblers. Psychology of Addictive Behaviors.

[CR26] Heath R, Brandt D, Nairn A (2006). Brand relationships: Strengthened by emotion, weakened by attention. Journal of Advertising Research.

[CR27] Hing N, Cherney L, Blaszczynski A, Gainsbury SM, Lubman DI (2014). Do advertising and promotions for online gambling increase gambling consumption? An exploratory study. International Gambling Studies.

[CR28] Hing N, Lamont M, Vitartas P, Fink E (2015). Sports-embedded gambling promotions: A study of exposure, sports betting intention and problem gambling amongst adults. International Journal of Mental Health and Addiction.

[CR29] Hing N, Russell AMT, Lamont M, Vitartas P (2017). Bet anywhere, anytime: An analysis of Internet sports bettors’ responses to gambling promotions during sports broadcasts by problem gambling severity. Journal of Gambling Studies.

[CR30] Hing N, Sproston K, Brook K, Brading R (2017). The structural features of sports and race betting inducements: Issues for harm minimisation and consumer protection. Journal of Gambling Studies.

[CR31] IGRG (2015). Gambling industry code for socially responsible advertising.

[CR32] Jensen K, Collins S (2008). The third-person effect in controversial product advertising. American Behavioral Scientist.

[CR33] Johnston MA, Bourgeois LR (2015). Third-person perceptions of gambling sponsorship advertising. Sport, Business and Management: An International Journal.

[CR34] Jonsson J, Rönnberg S, Meyer G, Hayer T, Griffiths M (2009). Sweden. Problem gambling in Europe: Challenges, prevention, and interventions.

[CR35] Lemarié L, Chebat J-C (2013). Resist or comply: Promoting responsible gambling among youth. Journal of Business Research.

[CR36] Lotteriinspektionen. (2015). *Unga möts av massiv marknadsföring kring spel om pengar* [Young people face massive marketing of gambling]. Retrieved from https://www.lotteriinspektionen.se/press/nyhetsarkiv/unga-mots-av-massiv-marknadsforing-kring-spel-om-pengar/

[CR37] McMullan JL, Miller D (2010). Advertising the “New fun-tier”: Selling casinos to consumers. International Journal of Mental Health and Addiction.

[CR38] Pallesen, S., Molde, H., Mentzoni, R. A., Hanss, D., & Morken, A. M. (2016). *Omfang av penge*- *og dataspillproblemer i Norge 2015* [The prevalence of problem gambling and problem gaming in Norway 2015]. Bergen: Universitetet i Bergen.

[CR39] Parke A, Harris A, Parke J, Rigbye J, Blaszczynski A (2015). Responsible marketing and advertising in gambling: A critical review. Journal of Gambling Business and Economics.

[CR40] Planzer S, Wardle H (2011). The comparative effectiveness of regulatory approaches and the impact of advertising on propensity for problem gambling.

[CR41] Romild U, Volberg R, Abbott M (2014). The Swedish Longitudinal Gambling Study (Swelogs): Design and methods of the epidemiological (EP-) track. International Journal of Methods in Psychiatric Research.

[CR42] Schottler Consulting (2012). The marketing, advertising and sponsorship of gambling products and services within New Zealand.

[CR43] Williams, R. J., West, B. L., & Simpson, R. I. (2012). *Prevention of problem gambling: A comprehensive review of the evidence, and identified best practices*. Report prepared for the Ontario Problem Gambling Research Centre and the Ontario Ministry of Health and Long Term Care. October 1, 2012.

[CR44] Zangeneh M, Griffiths MD, Parke J, Zangeneh M, Blaszczynski A, Turner N (2008). The marketing of gambling. In the pursuit of winning: Problem gambling theory, research and treatment.

